# Reconstructing phylogenies from noisy quartets in polynomial time with a high success probability

**DOI:** 10.1186/1748-7188-3-1

**Published:** 2008-01-24

**Authors:** Gang Wu, Ming-Yang Kao, Guohui Lin, Jia-Huai You

**Affiliations:** 1 Department of Computing Science, University of Alberta, Edmonton, Alberta T6G 2E8, Canada; 2 Department of Electrical Engineering and Computer Science, Northwestern University, Evanston, IL 60208, USA

## Abstract

**Background:**

In recent years, quartet-based phylogeny reconstruction methods have received considerable attentions in the computational biology community. Traditionally, the accuracy of a phylogeny reconstruction method is measured by simulations on synthetic datasets with known "true" phylogenies, while little theoretical analysis has been done. In this paper, we present a new model-based approach to measuring the accuracy of a quartet-based phylogeny reconstruction method. Under this model, we propose three efficient algorithms to reconstruct the "true" phylogeny with a high success probability.

**Results:**

The first algorithm can reconstruct the "true" phylogeny from the input quartet topology set without quartet errors in *O*(*n*^2^) time by querying at most (*n *- 4) log(*n *- 1) quartet topologies, where *n *is the number of the taxa. When the input quartet topology set contains errors, the second algorithm can reconstruct the "true" phylogeny with a probability approximately 1 - *p *in *O*(*n*^4 ^log *n*) time, where *p *is the probability for a quartet topology being an error. This probability is improved by the third algorithm to approximately $\frac{1}{1+q^{2}+\frac{1}{2}q^{4}+\frac{1}{16}q^{5}}$, where $q=\frac{p}{1-p}$, with running time of *O*(*n*^5^), which is at least 0.984 when *p *< 0.05.

**Conclusion:**

The three proposed algorithms are mathematically guaranteed to reconstruct the "true" phylogeny with a high success probability. The experimental results showed that the third algorithm produced phylogenies with a higher probability than its aforementioned theoretical lower bound and outperformed some existing phylogeny reconstruction methods in both speed and accuracy.

## Background

Evolution is a basic process in biology. The evolutionary history, referred to as *phylogeny*, of a set of taxa can be mathematically defined as a tree where the leaves are labeled with the given taxa and the internal nodes represent extinct or hypothesized ancestors. There are rooted and unrooted phylogenies. In a *rooted *phylogeny, an edge specifies the parent-child relationship and the root represents a common ancestor of all the taxa. A rooted phylogeny is called *binary *or *resolved *if every internal node has exactly two children. In an *unrooted *phylogeny, there is no parent-child relationship specified for an edge; and it is called *binary *or *resolved *if every internal node has degree exactly 3.

There have been many works on how to reconstruct rooted and unrooted phylogenies [1-3]. It is already known that rooted phylogenies and unrooted phylogenies can be transformed into each other [4], for example, by using an outgroup. In the remainder of this paper, a phylogeny refers to an unrooted binary phylogeny unless explicitly specified otherwise.

**Figure 1:**
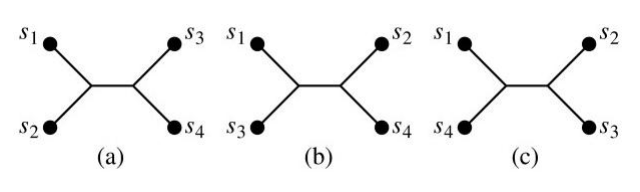
The three possible quartet topologies for quartet {*s*_1_, *s*_2_, *s*_3_, *s*_4_}.

Given a taxon set *S*, each subset of four taxa of *S *is called a *quartet *of *S*. In recent years, quartet-based phylogeny reconstruction methods have received considerable attentions in the computational biology community. In comparison with other phylogeny reconstruction methods, an advantage of quartet-based methods is that they can overcome the data disparity problem [5]. An unrooted phylogeny (or topology) of a quartet is called its *quartet topology*. Given a quartet {*s*_1_, *s*_2_, *s*_3_, *s*_4_} of *S*, there are three possible topologies associated with it, up to symmetry. These three quartet topologies are shown in Figure 1. For simplicity, we use [*s*_1_, *s*_2_|*s*_3_, *s*_4_] to denote the quartet topology in which the path connecting *s*_1 _and *s*_2 _does not intersect the path connecting *s*_3 _and *s*_4 _(see Figure 1(a)). The other two quartet topologies are [*s*_1_, *s*_3_|*s*_2_, *s*_4_] and [*s*_1_, *s*_4_|*s*_2_, *s*_3_].

Given a taxon set *S *and a phylogeny *T *on *S*, we can see that trimming all the other nodes (including the root if *T *is rooted) from *T *gives exactly one topology for every quartet of *S*. The quartet-based phylogeny reconstruction works inversely to first build a phylogeny for every quartet and then infer an overall phylogeny for the whole set of taxa. Suppose that *Q *is the set of quartet topologies built in the first step of a quartet-based phylogeny reconstruction, which can be done by various quartet inference methods [6-8]. If there exists a phylogeny *T *such that a quartet topology *q *in *Q *is the same as the one derived from *T*, then we say that *T satisfies q*, and *q *is *consistent *with *T*. If there exists a phylogeny *T *satisfying all quartet topologies in *Q*, then we say that *Q *is *compatible *and *T *is the (unique) phylogeny *associated *with *Q*. In the ideal case where all quartet topologies are "correct," *i.e*., *Q *is compatible, the task of assembling an overall phylogeny is easy and can be done in *O*(*n*^4^) time [9], where *n *is the number of taxa under consideration. In practice, however, some quartet topologies may be erroneous. Therefore, the set of quartet topologies may contain conflicting quartet topologies. This possibility complicates the overall quartet-based phylogeny reconstruction and presents an interesting computational challenge.

Given a taxon set *S*, we define the phylogeny that reveals the correct relationships among the taxa in *S *as the *"true" phylogeny *on *S*, denoted as *T*_true_. The *accuracy *of a phylogeny reconstruction method is the extent to which the generated phylogeny agrees with the "true" phylogeny. In many applications, the "true" phylogeny is not available to us for real-life instances in the study of evolution. Therefore, to investigate the accuracy of different reconstruction methods, synthetic data are created with simulations using a given evolutionary model, where the "true" phylogeny is known. If a quartet topology *q *∈ *Q *conflicts with *T*_true_, then *q *is a *quartet error*. Given a quartet topology set containing possible quartet errors, current phylogeny reconstruction methods seek to estimate the "true" phylogeny in one of the following two ways: (1) by a specific algorithm that leads to the determination of a phylogeny; or (2) by defining a measurement for the quality of generated phylogenies and searching for an optimal phylogeny. Purely algorithmic methods in the first category integrate phylogeny reconstruction and the definition of the preferred phylogeny tightly. These methods include quartet puzzling [10], the short quartet method [8], and semi-definite programming [4]. The methods in the first category tend to be computationally fast because they proceed directly toward the final solution without the evaluation of a large number of competing phylogenies. However, they can achieve high accuracy only on some specific datasets. Other statistical methods such as bootstrapping [11] are incorporated to assess the confidence of a found phylogeny, which requires extra computational time but may generate better phylogenies. These statistical methods have their limitations and may fail in some situations [12].

The second category of methods first define a score for each given quartet topology and then use combinatorial algorithms to find a phylogeny that achieves the optimal score. For example, the Maximum Quartet Consistency (MQC) problem [13], which is NP-hard, aims to compute a phylogeny which respects as many quartet topologies as possible. Several attempts have been made to solve MQC optimally [5,14,15] or approximately [16,17]. The *hypercleaning *algorithm proposed in [18] aims to reconstruct a phylogeny that minimizes a certain quartet distance value for measuring the quartet errors. The complexity of the hypercleaning algorithm is *O*(*n*^5 ^*f*(2*m*) + *n*^7 ^*f*(*m*)), where *f*(*m*) = 4*m*^2^(1 + 2*m*)^4*m*^, *n *is the number of taxa, and *m *is a value based on the quartet distance model. These methods tend to be much slower than those in the first category but have higher accuracy. For datasets with a relatively large number of quartet errors, the optimal phylogenies produced by these methods may not be unique, and one must provide additional measurements to estimate the "true" phylogeny.

Traditionally, the performance accuracy of a phylogeny reconstruction method is measured by simulations on synthetic datasets with a known "true" phylogeny, while little theoretical analysis has been done. In this paper, we propose a new model-based approach to measuring the accuracy of a quartet-based phylogeny reconstruction method, *i.e*., to analyze the probability of reconstructing the "true" phylogeny.

## Methods

We define our data model and describe our three phylogeny reconstruction algorithms in this section.

### Probabilistic model of quartet generation

In this section, we define a probabilistic model for the quartet-based phylogeny reconstruction and introduce some terminologies that will be used in the discussion of three new algorithms.

Given a quartet topology set *Q *on a taxon set *S *= {*s*_1_, *s*_2_,...,*s*_*n*_}, *Q *is *complete *if *Q *contains exactly one quartet topology for every quartet of *S*. In this paper, we assume *Q *is complete. Given a phylogeny *T *on a taxon set *S *= {*s*_1_, *s*_2_,...,*s*_*n*_}, *n *is the *size *of *T*, and we use *Q*_*T *_to denote the complete quartet topology set induced by *T*. Given *T*_true_, our simulation model first generates a complete quartet topology set $Q_{T_{\text{true}}}$ for *T*_true_. For every quartet topology in $Q_{T_{\text{true}}}$, with probability 1 - *p *(0 ≤ *p *≤ 1) our simulation model does not do anything to it, and with probability $\frac{p}{2}$ changes its topology into each of the other two topologies. In this way, the model generates the input quartet topology set *Q*, and consequently every quartet topology in the generated set *Q *has the same probability *p *of being a quartet error. This probability *p *is called the *quartet error probability *associated with the instance. Under this model, our main computational objective is to reconstruct *T*_true _from *Q *with a high success probability while minimizing the time complexity.

In practice, the quartet error probability *p *mainly depends on the quality of the quartet inference methods, such as the Four-point method [9], the Neighbor Joining method [6], and the Ordinal Quartet method [7]. Simulation results in [7] show that the Ordinal Quartet method can achieve over 80% accuracy while inferring quartet topologies. Therefore, in our model we assume that current quartet inference methods can infer more correct quartet topologies than erroneous ones. In particular, we assume the quartet error probability 0 ≤ *p *<$\frac{1}{3}$. As this paper focuses on phylogeny reconstruction, we also assume that the time complexity of inferring one quartet topology is *O*(1).

### An *O*(*n*^2^)-time algorithm for reconstructing *T*_true _when *p *= 0

In this section, we assume that no quartet errors exist in *Q*. Our algorithm is based on the following classic result by Jordan [19].

**Lemma 1 (see **[19]**) ***Given a tree T with n leaves, there exists an internal node whose removal partitions the tree into connected components, each with at most *$\frac{n}{2}$*leaves, and such a node can be found in linear time*.

Given an unrooted binary phylogeny *T*, if we remove an internal node *v *from *T*, *T *will be divided into three sub-phylogenies. We denote these three sub-phylogenies as *T *- {*v*}. Based on Lemma 1, there exists an internal node *v *in *T *such that each of the trees in *T *- {*v*} has at most $\frac{n}{2}$ leaves. An internal node *v *of *T *having such a property is called a *separator *of *T*. Notice that a phylogeny *T *may have more than one separator, but our algorithms in Tables 1, 2, and 3 need only one of them. Given a phylogeny *T *and a separator *v *of *T*, we can merge two sub-phylogenies of *T *- {*v*} into one leaf node (replacing the separator *v*), which is treated as a *super taxon *to represent the union of the taxon sets of the two merged sub-phylogenies.

**Table 1:** 

Q-RAND(*S*, *Q*):	
1.	Randomly select a quartet topology in *Q *as the initial phylogeny *T*;
2.	Delete the four taxa of *T *from the taxon set *S*;
3.	Randomly select a taxon *s *from *S*;
4.	Locate a separator *v *of *T*;
5.	Randomly select a taxon from each sub-phylogeny of *T *- {*v*}, say *s*_*a*_, *s*_*b*_, and *s*_*c*_;
6.	Decide which sub-phylogeny of *T *- {*v*} taxon *s *should be inserted into based on the quartet topology for {*s*_*a*_, *s*_*b*_, *s*_*c*_, *s*};
7.	If the located sub-phylogeny has only one edge,
7.1.	Insert *s *on that edge and let the new phylogeny be *T*;
8.	Else,
8.1.	Merge the other two sub-phylogenies as a super taxon (which replaces *v*);
8.2.	Let the located sub-phylogeny with the super taxon be the new current phylogeny *T*;
8.3.	Go back to Step 4;
9.	Delete taxon *s *from *S*;
10.	If *S *is not empty,
10.1.	Go back to Step 3;
11.	Else,
11.1.	Output the phylogeny *T*.

**Table 2:** 

Q-VOTE(*S*, *Q*, *p*):	
1.	Randomly select a quartet topology in *Q *as the initial phylogeny *T*;
2.	Delete the four taxa of *T *from the taxon set *S*;
3.	Randomly select a taxon *s *from *S*;
4.	Locate a separator *v *of *T*;
5.	Decide which sub-phylogeny of *T *- {*v*} taxon *s *should be inserted into based on the votes;
6.	If the located sub-phylogeny has only one edge,
6.1.	Insert taxon *s *on that edge and let the new phylogeny be *T*;
7.	Else,
7.1.	Merge the other two sub-phylogenies as a super taxon (which replaces *v*);
7.2.	Let the located sub-phylogeny with the super taxon be the new current phylogeny *T*;
7.3.	Go back to Step 4;
8.	Delete taxon *s *from *S*;
9.	If *S *is not empty,
9.1.	Go back to Step 3;
10.	Else,
10.1.	Output the phylogeny *T*.

**Table 3:** 

M-VOTE(*S*, *Q*, *p*):	
1.	Search for a 5-subset compatible with *Q*;
2.	If successful
2.1	Let the corresponding phylogeny be the current phylogeny *T*;
2.2	Delete the 5 taxa of *T *from the taxon set *S*;
3.	Else
3.1	Randomly select a quartet topology in *Q *as the current phylogeny *T*;
3.2	Delete the four taxa of *T *from the taxon set *S*;
4.	Randomly select a taxon *s *from *S*;
5.	Locate a separator *v *of *T*;
6.	Decide which sub-phylogeny of *T *- {*v*} taxon *s *should be inserted into based on the votes;
7.	If the located sub-phylogeny has only one edge,
7.1.	Insert taxon *s *on that edge and let the new phylogeny be *T*;
8.	Else,
8.1.	Merge the other two sub-phylogenies as a super taxon (which replaces *v*);
8.2.	Let the located sub-phylogeny with the super taxon be the new current phylogeny *T*;
8.3.	Go back to Step 5;
9.	Delete taxon *s *from *S*;
10.	If *S *is not empty,
10.1.	Go back to Step 4;
11.	Else,
11.1.	Output the phylogeny *T*.

Given a quartet topology set *Q *with no quartet errors, we can start with a randomly selected quartet topology *q*, which forms an initial phylogeny *T*_4 _on 4 taxa, and then iteratively insert a new taxon to grow the phylogeny. To ensure that the true phylogeny on the whole taxon set is recovered, in the *i*-th iteration to insert taxon *s*_*i*+4_, we first locate a separator, *v*, of phylogeny *T*_*i*+3_. Then, we randomly select a taxon from each of the three sub-phylogenies of *T*_*i*+3 _- {*v*}. Suppose that these three selected taxa are *s*_*a*_, *s*_*b*_, and *s*_*c*_. We proceed to check the given topology in *Q *on quartet {*s*_*a*_, *s*_*b*_, *s*_*c*_, *s*_*i*+4_}. Based on that topology, we can determine which sub-phylogeny taxon *s*_*i*+4 _should be inserted into. For example, if the topology is [*s*_*a*_, *s*_*b*_|*s*_*c*_, *s*_*i*+4_], then *s*_*i*+4 _should be inserted into the sub-phylogeny that contains *s_c_*as its leaf. Recursively, we treat the other two sub-phylogenies as a super taxon (which replaces the separator *v*) on the located sub-phylogeny to generate a new phylogeny, and to determine the location in this new phylogeny where taxon *s*_*i*+4 _should be inserted. A high-level description of this algorithm Q-RAND is summarized in Table 1.

**Theorem 2 ***Given a quartet topology set Q with no quartet errors, T*_true _*can be constructed in O*(*n*^2^) *time by querying at most *(*n *- 4) log(*n *- 1) *quartet topologies in Q*.

PROOF. The Q-RAND algorithm described above and detailed in Table 1 can be employed to construct the true phylogeny, where one can easily see that the final phylogeny obtained after inserting all the taxa satisfies all the quartet topologies in *Q*, and therefore it is *T*_true_.

In the *i*-th iteration, Q-RAND needs to query at most log(*i *+ 3) quartet topologies. Therefore, the total number of quartet topologies need to be queried is at most log 4 + log 5 + ⋯ + log(*n *- 1) ≤ (*n *- 4) log(*n *- 1). As we only need *O*(1) time to infer each queried quartet topology, the time complexity of querying these quartet topologies is *O*(*n *log *n*).

Based on Lemma 1, finding a separator of phylogeny *T*_*i *_takes *O*(*i*) time. Thus the time of finding the separators during the *i*-th iteration is *O*(*i *+ *i*/2 + ⋯ + 1) = *O*(*i*). The overall time of Q-RAND is therefore *O*(*n*^2^).   □

An *experiment *is a rooted phylogeny on three taxa. There has been extensive work on reconstructing phylogenies from a set of experiments with no errors. In general, there is a trade-off between the number of queried experiments and the running time. Kannan *et al*. [20] gave an Ω(*n *log *n*) lower bound of queried experiments for reconstructing rooted binary phylogenies in *O*(*n*^2^) time. Kao *et al*. [21] presented a randomized algorithm with running time *O*(*n *log *n *log log *n*) using *O*(*n *log *n *log log *n*) experiments. The fastest algorithm [22] so far is a deterministic algorithm which can reconstruct the true phylogeny in *O*(*n *log *n*) time by querying at most *n*(log *n *+ *O*(1)) experiments. Although these algorithms and complexity results are for reconstructing phylogenies from experiments, they also apply to quartet-based phylogeny reconstruction through straightforward transformation. Therefore, algorithm Q-RAND achieves the lower bound of queried quartet topologies for phylogeny reconstruction from a given quartet topology set without errors. Q-RAND will be the base structure of our algorithms for the case with quartet errors.

### Reconstructing *T*_true _with a high success probability when 0 <*p *<$\frac{1}{3}$

If the input quartet topology set *Q *contains quartet errors, then algorithm Q-RAND may make a wrong decision while locating the sub-phylogeny where taxon *s*_*i *_should be inserted. In this section, we address this issue by adding a voting scheme to algorithm Q-RAND to aggregate the information in the correct quartet topologies. The key observation is that, when *p *is small, in order to incorrectly identify the location for a new taxon, there must exist many quartet errors among the queried quartet topologies that all support the decision, which however is unlikely.

The new algorithm is called Q-VOTE, which also starts with an randomly picked quartet topology. In the *i*-th iteration to insert taxon *s*_*i*+4_, the algorithm first locates a separator, *v*, of phylogeny *T*_*i*+3_. It then queries all the possible quartet topologies on {*s*_*a*_, *s*_*b*_, *s*_*c*_, *s*_*i*+4_}, where *s*_*a*_, *s*_*b*_, and *s*_*c *_come from the taxon sets of the three sub-phylogenies of *T*_*i*+3 _- {*v*}, respectively. If a sub-phylogeny contains a super taxon, which is formed by merging two sub-phylogenies in a previous step, all the taxa represented by that super taxon are also taken into consideration. Suppose that the taxon sets of the three sub-phylogenies have sizes *m*_1_, *m*_2_, and *m*_3_, respectively. Then there are *m*_1 _× *m*_2 _× * m*_3 _quartet topologies that we need to consider. Each quartet topology gives a *vote *for a sub-phylogeny into which taxon *s*_*i*+4 _should be inserted. For example, the quartet topology [*s*_*a*_, *s*_*b*_|*s*_*c*_, *s*_*i*+4_] gives a vote on the sub-phylogeny whose taxon set includes *s*_*c*_. The algorithm then chooses the sub-phylogeny that has the maximum votes and recursively calls the above procedure until the location of taxon *s*_*i*+4 _is determined. We call each recursive step described above a *decision *to locate taxon *s*_*i*+4_. In each decision, the algorithm needs to query *O*(*i*^3^) quartet topologies, and log *i *decisions are needed to determine the final location of taxon *s*_*i*+4_. Therefore, the overall running time of algorithm Q-VOTE is *O*(*n*^4 ^log *n*). A high-level description of algorithm Q-VOTE is summarized in Table 2.

**Theorem 3 ***When *0 <*p *<$\frac{1}{3}$, *algorithm Q-VOTE can reconstruct T*_true _*in O*(*n*^4 ^log *n*) *time with a probability at least *$(1-p)\prod_{j=4}^{n-1}{\left[1-\sum_{k=\frac{j-2}{2}}^{j-2}\left(\begin{array}{c} j-2\\ k \end{array}\right)p^{k}{\left(1-p\right)}^{j-2-k}\right]}^{\log j}$, *where n is the size of the input taxon set and p is the quartet error probability of the input quartet topology set*.

PROOF. Suppose that the algorithm queries *N *quartet topologies when it makes one decision of locating taxon *s*_*j*+1 _on a phylogeny *T*_*j *_with *j *taxa. It is easy to see that *N *≥ *j *- 2. The algorithm makes a wrong decision only if the number of quartet errors among these queried quartet topologies is at least $\frac{N}{2}$. (Note that, however, the existence of at least $\frac{N}{2}$ quartet errors does not necessarily imply the misplacement of taxon *s*_*j*+1_.) We know that each quartet topology has a probability *p *to be a quartet error. Therefore, the number of quartet errors follows a binomial distribution, and the probability that the algorithm makes a wrong decision is at most

$$\sum_{k=\frac{N}{2}}^{N}\left(\begin{array}{c} N\\ k \end{array}\right)p^{k}{\left(1-p\right)}^{N-k}\leq \sum_{k=\frac{j-2}{2}}^{j-2}\left(\begin{array}{c} j-2\\ k \end{array}\right)p^{k}{\left(1-p\right)}^{j-2-k},$$

(The detailed proof of this inequality is provided in Appendix A.)

Since the algorithm makes log *j *decisions to locate the final position of taxon *s*_*j*+1_, the probability that the algorithm locates the correct position for taxon *s*_*j*+1 _is at least

$${\left[1-\sum_{k=\frac{j-2}{2}}^{j-2}\left(\begin{array}{c} j-2\\ k \end{array}\right)p^{k}{\left(1-p\right)}^{j-2-k}\right]}^{\log j}.$$

Therefore, the algorithm can construct *T*_true _with a probability at least

$$\left(1-p\right)\prod_{j=4}^{n-1}{\left[1-\sum_{k=\frac{j-2}{2}}^{j-2}\left(\begin{array}{c} j-2\\ k \end{array}\right)p^{k}{\left(1-p\right)}^{j-2-k}\right]}^{\log j}.$$

The first term, 1 - *p*, is the probability that the algorithm chooses a correct starting quartet topology.

### Improvements

We can see that the maximum probability of algorithm Q-VOTE to make a wrong decision, $\sum_{k=\frac{j-2}{2}}^{j-2}\left(\begin{array}{c} j-2\\ k \end{array}\right)p^{k}{\left(1-p\right)}^{j-2-k}$, is close to 0, when *j *is relatively large. Therefore, the probability that the algorithm can reconstruct *T*_true _mainly depends on the correctness of the phylogeny with the first several inserted taxa. Based on this observation, we propose the following improvement to algorithm Q-VOTE to look for a good starting phylogeny that contains *m *taxa for *m *≥ 4.

Given a taxon set *S*, each subset of *m *(*m *≥ 4) taxa of *S *is called an *m-subset *of *S*. A quartet topology is *associated *with an *m*-subset if the four taxa of the quartet topology are all in the *m*-subset. An *m*-subset is *compatible *with *Q *if the set of its associated quartet topologies in *Q *is compatible. It is easy to see that a compatible *m*-subset has exactly one topology, which can be constructed from its associated quartet topologies in *Q*.

In the following, we only consider *m *= 5, while our conclusion can be generalized to larger *m *with increased running time. The new algorithm, called M-VOTE, first goes through all the possible 5-subsets to find a compatible 5-subset. If successful, M-VOTE starts with the phylogeny on the compatible 5-subset and proceeds as Q-VOTE to insert all the other taxa into the phylogeny one by one. If unsuccessful, M-VOTE starts with a randomly selected quartet topology, and it reduces to Q-VOTE. A high-level description of algorithm M-VOTE is summarized in Table 3.

**Theorem 4 ***When *0 <*p *<$\frac{1}{3}$*and Step 1 of algorithm M-VOTE is successful, then the algorithm can reconstruct T*_true _*in O*(*n*^5^) *time with a probability at least*

$$\left[\frac{1}{1+q^{2}+\frac{1}{2}q^{4}+\frac{1}{16}q^{5}}\right]\cdot \prod_{j=5}^{n-1}{\left[1-\sum_{k=\frac{j-2}{2}}^{j-2}\left(\begin{array}{c} j-2\\ k \end{array}\right)p^{k}{\left(1-p\right)}^{j-2-k}\right]}^{\log j},$$

*where n is the size of the input taxon set*, $q=\frac{p}{1-p}$, *and p is the quartet error probability of the input quartet topology set*.

PROOF. Finding a compatible 5-subset needs *O*(*n*^5^) time. In each iteration of inserting a taxon into the current phylogeny, the algorithm goes through all the remaining taxa to make a selection. Therefore the overall running time of the algorithm is $O(n^{5}+\sum_{i=5}^{n-1}i^{3}(n-i)\log i)=O(n^{5}+n^{4}\log n)=O(n^{5})$.

Suppose that in Step 1 the phylogeny constructed from the compatible 5-subset is *T*_5 _and the true phylogeny of this 5-subset is ${T^{\prime }}_{5}$. Note that there are 15 possible phylogenies on this 5-subset, including ${T^{\prime }}_{5}$ itself. If *T*_5 _≠ ${T^{\prime }}_{5}$, then it is easy to see that $\left|Q_{T_{5}}-Q_{{T^{\prime }}_{5}}\right|$ = 2, 4, or 5.

Under the assumption that every quartet topology has probability *p *to be erroneous, we show in the following that $\left|Q_{T_{5}}-Q_{{T^{\prime }}_{5}}\right|$ has different probabilities to be 0, 2, 4, and 5 (but no probability to be 1 or 3).

First of all, clearly, $\left|Q_{T_{5}}-Q_{{T^{\prime }}_{5}}\right|$ = 0 as probability (1 - *p*)^5^, since every one of the 5 quartet topologies has to be correct. For each phylogeny *T*_5 _such that $\left|Q_{T_{5}}-Q_{{T^{\prime }}_{5}}\right|$ = 2, *i.e*., there are two quartet errors, we conclude that these two quartet errors must contain a common subset of three taxa out of the five, and the induced sub-phylogeny of ${T^{\prime }}_{5}$ on these three taxa should not contain any other taxon from the five. Since the probability to observe *T*_5 _is $\frac{1}{4}p^{2}{\left(1-p\right)}^{3}$ and there are exactly four possible topologies for *T*_5_, $\left|Q_{T_{5}}-Q_{{T^{\prime }}_{5}}\right|$ = 2 has probability 4 × $\frac{1}{4}p^{2}{\left(1-p\right)}^{3}$. A similar analysis shows that there are eight possible *T*_5_'s such that $\left|Q_{T_{5}}-Q_{{T^{\prime }}_{5}}\right|$ = 4, and $\left|Q_{T_{5}}-Q_{{T^{\prime }}_{5}}\right|$ = 4 has probability $8\times \frac{1}{16}p^{4}\left(1-p\right)$; there are two possible *T*_5_'s such that $\left|Q_{T_{5}}-Q_{{T^{\prime }}_{5}}\right|$ = 5, and $\left|Q_{T_{5}}-Q_{{T^{\prime }}_{5}}\right|$ = 5 has probability $2\times \frac{1}{32}p^{5}$.

To summarize, the probability of observing incorrect phylogenies on this 5-subset is

$$p^{2}{\left(1-p\right)}^{3}+\frac{1}{2}p^{4}\left(1-p\right)+\frac{1}{16}p^{5},$$

and thus the probability of obtaining a phylogeny *T*_5 _and *T*_5 _= ${T^{\prime }}_{5}$ is

$$\frac{{\left(1-p\right)}^{5}}{{\left(1-p\right)}^{5}+p^{2}{\left(1-p\right)}^{3}+\frac{1}{2}p^{4}\left(1-p\right)+\frac{1}{16}p^{5}}=\frac{1}{1+q^{2}+\frac{1}{2}q^{4}+\frac{1}{16}q^{5}},$$

where $q=\frac{p}{1-p}<\frac{1}{2}$ (and the success probability is greater than 0.779) when 0 <*p *<$\frac{1}{3}$. After the 5-subset is identified, M-VOTE proceeds as Q-VOTE and therefore it can construct *T*_true _with a probability at least

$$\left[\frac{1}{1+q^{2}+\frac{1}{2}q^{4}+\frac{1}{16}q^{5}}\right]\cdot \prod_{j=5}^{n-1}{\left[1-\sum_{k=\frac{j-2}{2}}^{j-2}\left(\begin{array}{c} j-2\\ k \end{array}\right)p^{k}{\left(1-p\right)}^{j-2-k}\right]}^{\log j}.$$

Notice that to increase the success probability, Step 1 of algorithm M-VOTE can be changed to search for a compatible *m*-subset for any *m > *5. Furthermore, if the search is not successful, then the algorithm can look for a compatible (*m *- 1)-subset, and so on. In the worst case, the starting phylogeny is a randomly selected quartet topology, which has 1 - *p *probability not to be an error. In the following lemma, we show that if the number of quartet errors is not too large or the quartet error probability *p *is small, then we can almost always find a compatible *m*-subset for *m *≥ 5.

**Lemma 5 ***Given a quartet topology set Q with k quartet errors, there exists at least one compatible m-subset if *$k<\frac{\left|Q\right|}{\left(\begin{array}{c} m\\ 4 \end{array}\right)}$, *where m *≥ 5.

PROOF. Given an *m*-subset {*s*_1_, *s*_2_,...,*s*_*m*_}, there are $\left(\begin{array}{c} m\\ 4 \end{array}\right)$ quartet topologies in *Q *that are associated with it. If the set of these $\left(\begin{array}{c} m\\ 4 \end{array}\right)$ quartet topologies is not compatible, then there must exist at least one quartet error in it. Since a quartet topology is associated with exactly $\left(\begin{array}{c} n-4\\ m-4 \end{array}\right)$*m*-subsets, the total number of *m*-subsets associated with at least one quartet error is at most $\left(\begin{array}{c} n-4\\ m-4 \end{array}\right)k<\frac{\left(\begin{array}{c} n-4\\ m-4 \end{array}\right)\left(\begin{array}{c} n\\ 4 \end{array}\right)}{\left(\begin{array}{c} m\\ 4 \end{array}\right)}=\left(\begin{array}{c} n\\ m \end{array}\right)$. Note that there are $\left(\begin{array}{c} n\\ m \end{array}\right)$*m*-subsets. Therefore, at least one *m*-subset is compatible.   □

**Figure 2:**
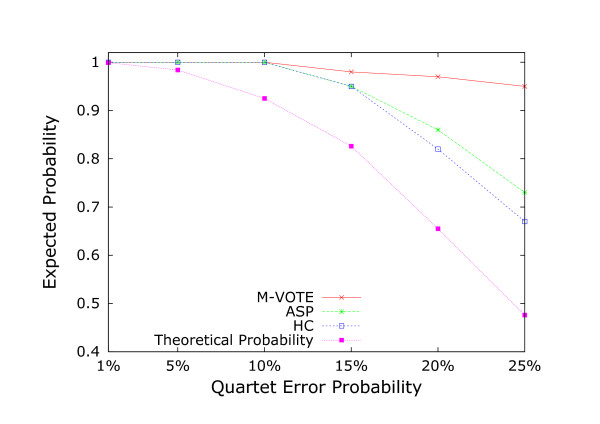
Probability comparison among the proposed algorithm M-VOTE, the hypercleaning algorithm (HC), the answer set programming method for the MQC problem (ASP), and the theoretical success probability of M-VOTE from Theorem 4.

Given a quartet error probability *p*, the expected number of quartet errors in *Q *is *p*|*Q*|. It follows from Lemma 5 that if $p<\frac{1}{\left(\begin{array}{c} m\\ 4 \end{array}\right)}$, then there is a high probability for the existence of a compatible *m*-subset. For instance, when *p *< 0.05, algorithm M-VOTE almost always find a compatible 5-subset (and the probability that the associated phylogeny is correct is at least 0.984; see Figure 2).

### Experimental results

To investigate the practical performance of algorithm M-VOTE, we performed experiments on a set of synthetic data. For a set *S *of *n *taxa, we generated a phylogeny by recursively joining randomly selected subtrees. The subtrees were selected from a set that initially only contained the one-node subtrees each corresponding to a given taxon. When two subtrees were joined, we replaced them in the set by the newly generated subtree. The resulting phylogeny on *n *taxa was treated as the "true" phylogeny *T*_true_. A complete quartet topology set, denoted as $Q_{T_{\text{true}}}$, was then induced by this phylogeny. For every quartet on *S*, we altered its topology in $Q_{T_{\text{true}}}$ by a probability *p *(0 <*p *<$\frac{1}{3}$) into a topology randomly selected from the other two possible topologies for the quartet. We treated the altered quartet topologies as quartet errors and the resulting quartet topology set as the input to the algorithms in our experiments. Each generated dataset is labeled by a pair (*n*, *p*) where *n *is the number of taxa and *p *records the quartet error probability of the input complete quartet topology set. We used the quartet error probability *p *= 1%, 5%, 10%, 15%, 20%, 25%, and the taxon set size *n *= 20, 25, 30, 35, 40, 45, 50. For every pair of (*n*, *p*), we generated 100 datasets. Therefore, given a quartet error probability *p*, we have 700 datasets associated with it. In our experiments, we compared our proposed algorithm M-VOTE with the hypercleaning algorithm (HC) [18], and the answer set programming method (ASP) for the MQC problem [15] in terms of the probability to construct "true" phylogenies.

Given a dataset *D *and an algorithm *A*, let the phylogeny constructed by algorithm *A *from *D *be *T*_*D *_and the "true" phylogeny of *D *be *T*_true_. If $\left|Q_{L_{D}}-Q_{T_{\text{true}}}\right|$ = 0, then we say that dataset *D *can be correctly recovered by algorithm *A*. Given a probability value *p*, we applied each algorithm to the corresponding 700 datasets, and calculated the total number of datasets that could be correctly recovered, referred to as *c*. We then used $\frac{c}{700}$ as the expected probability of the algorithm to construct "true" phylogenies. In our experiments, we used the expected probability as a score to quantify the performance of the algorithms. In Figure 2, we compare the expected probability values of M-VOTE, HC, and ASP, and the theoretical success probability values based on Theorem 4. As shown in Figure 2, algorithm M-VOTE produced "true" phylogenies with the highest probability, and the probability values of algorithm M-VOTE were always higher than the theoretical ones. As the reported time complexity of the hyper-cleaning algorithm (*O*(*n*^5^*f*(2*m*) + *n*^7^*f*(*m*))) is much higher than that of our algorithm M-VOTE, and the ASP method is an exact method for the NP-hard MQC problem, M-VOTE is therefore the fastest and most accurate one.

## Discussion and Conclusions

In this paper, we have proposed an *O*(*n*^2^)-time algorithm (Q-RAND) to reconstruct a phylogeny from a quartet topology set without quartet errors. This algorithm achieves the optimal lower bound on the number of quartet topology queries. We have also proposed a probabilistic model for the quartet-based phylogeny reconstruction. Under this model, two algorithms (Q-VOTE and M-VOTE) are proposed to reconstruct a phylogeny on a quartet topology set with errors. These two algorithms are mathematically guaranteed to reconstruct the "true" phylogeny with high success probabilities. The key to our algorithms for being able to achieve a high success probability is that for making a wrong decision on the location of a new taxon, there must exist a large number of quartet errors among the queried quartet topologies, which is unlikely. Although we only showed that this is a small probability event under the binomial distribution, we believe that this should be a small probability event also under other probability distributions. The experimental results showed that algorithm M-VOTE produced "true" phylogenies with a higher probability than the theoretical success probability stated in Theorem 4, and it outperformed two existing phylogeny reconstruction methods in both speed and accuracy.

This work opens up several research directions. First of all, in real world phylogeny reconstruction, the distribution of quartet errors is largely unknown, both theoretically and empirically. The probabilistic model and algorithms proposed in this paper can be regarded as the first step toward reconstructing the "true" phylogeny with a high success probability. Csűrös and Kao [1] proposed an algorithm that can reconstruct the true phylogeny with a high probability in the Jukes-Cantor model of evolution [23]. Our next step would be to investigate possible probabilistic properties of the quartet topology set under some models of evolution and to design algorithms that can reconstruct the true phylogeny with a high probability under such evolutionary models. Secondly, it would be interesting to investigate the relationships between the accuracy of the reconstructed phylogeny and the topology of the true phylogeny. In general, the larger the quartet error probability *p *is, the more difficult it is to reconstruct the true phylogeny and therefore the lower the accuracy is. However, under the same quartet error probability, it is interesting to investigate whether different topologies of the true phylogeny may affect the accuracy of our algorithms. Thirdly, some computational questions are still open. Can we reduce the running time of the proposed algorithms by utilizing the techniques proposed in [20-22]? We know that there is a trade-off between the running time and the number of queried quartet topologies, as demonstrated in Theorem 4. If we attempt to reduce the running time by querying fewer quartet topologies, what is the success probability of the new algorithm to reconstruct the true phylogeny?

Appendix A

**Theorem 6 ***If N is an even number and *0 <*p *<$\frac{1}{3}$, *then*

$$\sum_{k=\frac{N}{2}}^{N}\left(\begin{array}{c} N\\ k \end{array}\right)p^{k}{\left(1-p\right)}^{N-k}\geq \sum_{k=\frac{N}{2}+1}^{N+1}\left(\begin{array}{c} N+1\\ k \end{array}\right)p^{k}{\left(1-p\right)}^{N+1-k}$$

and

$$\sum_{k=\frac{N}{2}}^{N}\left(\begin{array}{c} N\\ k \end{array}\right)p^{k}{\left(1-p\right)}^{N-k}\geq \sum_{k=\frac{N}{2}+1}^{N+2}\left(\begin{array}{c} N+2\\ k \end{array}\right)p^{k}{\left(1-p\right)}^{N+2-k}.$$

PROOF. For the first inequality,

$$\begin{array}{lll} \frac{k+1}{N+1}\geq p & \Leftrightarrow & \left(\begin{array}{c} N\\ k \end{array}\right)\geq \left(\begin{array}{c} N+1\\ k+1 \end{array}\right)p\\ & \Leftrightarrow & \left(\begin{array}{c} N\\ k \end{array}\right)p^{k}{\left(1-p\right)}^{N-k}\geq \left(\begin{array}{c} N+1\\ k+1 \end{array}\right)p^{k+1}{\left(1-p\right)}^{N-k}\\ & \Leftrightarrow & \sum_{k=\frac{N}{2}}^{N}\left(\begin{array}{c} N\\ k \end{array}\right)p^{k}{\left(1-p\right)}^{N-k}\geq \sum_{k=\frac{N}{2}+1}^{N+1}\left(\begin{array}{c} N+1\\ k \end{array}\right)p^{k}{\left(1-p\right)}^{N+1-k}. \end{array}$$

For the second inequality, it is easy to prove that

$$\begin{array}{c} \left(\begin{array}{c} N\\ \frac{N}{2} \end{array}\right)p^{\frac{N}{2}}{\left(1-p\right)}^{\frac{N}{2}}\geq \frac{9}{8}\left(\begin{array}{c} N+2\\ \frac{N}{2}+1 \end{array}\right)p^{\frac{N}{2}+1}{\left(1-p\right)}^{\frac{N}{2}+1},\\ \left(\begin{array}{c} N\\ \frac{N}{2}+1 \end{array}\right)p^{\frac{N}{2}+1}{\left(1-p\right)}^{\frac{N}{2}-1}\geq \left(\begin{array}{c} N+2\\ \frac{N}{2}+2 \end{array}\right)p^{\frac{N}{2}+2}{\left(1-p\right)}^{\frac{N}{2}},\\ \left(\begin{array}{c} N\\ \frac{N}{2}+2 \end{array}\right)p^{\frac{N}{2}+2}{\left(1-p\right)}^{\frac{N}{2}-2}\geq \left(\begin{array}{c} N+2\\ \frac{N}{2}+3 \end{array}\right)p^{\frac{N}{2}+3}{\left(1-p\right)}^{\frac{N}{2}-1},\\ \frac{1}{8}\left(\begin{array}{c} N+2\\ \frac{N}{2}+1 \end{array}\right)p^{\frac{N}{2}+1}{\left(1-p\right)}^{\frac{N}{2}+1}\geq \left(\begin{array}{c} N+2\\ \frac{N}{2}+4 \end{array}\right)p^{\frac{N}{2}+4}{\left(1-p\right)}^{\frac{N}{2}-2},\\ \left(\begin{array}{c} N\\ \frac{N}{2}+3 \end{array}\right)p^{\frac{N}{2}+3}{\left(1-p\right)}^{\frac{N}{2}-3}\geq \left(\begin{array}{c} N+2\\ \frac{N}{2}+5 \end{array}\right)p^{\frac{N}{2}+5}{\left(1-p\right)}^{\frac{N}{2}-3},\\ \vdots \\ p^{N}\geq p^{N+2}. \end{array}$$

Therefore,

$$\sum_{k=\frac{N}{2}}^{N}\left(\begin{array}{c} N\\ k \end{array}\right)p^{k}{\left(1-p\right)}^{N-k}\geq \sum_{k=\frac{N}{2}+1}^{N+2}\left(\begin{array}{c} N+2\\ k \end{array}\right)p^{k}{\left(1-p\right)}^{N+2-k}.$$

## Authors' contributions

All authors contributed equally to this work, and read and approved the final manuscript.
